# Acupuncture for pain caused by prostate cancer: Protocol for a systematic review

**DOI:** 10.1097/MD.0000000000013954

**Published:** 2019-01-11

**Authors:** Jisheng Wang, Yi Lei, Binghao Bao, Xudong Yu, Hengheng Dai, Fei Chen, Haisong Li, Bin Wang

**Affiliations:** a Graduate School of Beijing University of Chinese Medicine; b Department of Andrology, Dongzhimen Hospital, Beijing University of Chinese Medicine, Beijing; c Department of Andrology, The Second Affiliated Hospital of Shaanxi University of Traditional Chinese Medicine, Shaanxi, China.

**Keywords:** acupuncture, pain, protocol, prostate cancer, systematic review

## Abstract

**Background:**

Prostate cancer is a male malignant tumor disease with high prevalence in recent years. Patients with advanced prostate cancer are more likely to have bone metastasis and strong bone pain, and even lead to pathological fracture, which has a serious impact on the quality of life of patients. Acupuncture has good clinical efficacy in treating pain caused by prostate cancer. This review hopes to adopt meta-analysis to evaluate the efficacy and safety of acupuncture in the treatment of pain caused by prostate cancer and provides evidence for its application in clinical practice.

**Methods and analysis:**

We will search for PubMed, Cochrane Library, AMED, EMbase, WorldSciNet, Nature, Science online and China Journal Full-text Database (CNKI), China Biomedical Literature CD-ROM Database (CBM), and related randomized controlled trials included in the China Resources Database. The time is limited from the construction of the library to November 2018. We will use the criteria provided by Cochrane 5.1.0 for quality assessment and risk assessment of the included studies, and use the Revman 5.3 and Stata 13.0 software for meta-analysis of the effectiveness, recurrence rate, and symptom scores of epididymitis.

**Ethics and dissemination:**

This systematic review will evaluate the efficacy and safety of acupuncture for pain caused by prostate cancer. Owing to the fact that all of the data used in this systematic review and meta-analysis have been published, this review does not require ethical approval. Furthermore, all data will be anonymously analyzed during the review process trial.

**Trial registration number:**

PROSPERO CRD42018111550

## 1 Introduction

Prostate cancer is a male malignant tumor disease which has a high prevalence in recent years.^[1]^ Patients who suffer from advanced prostate cancer are vulnerable to osseous metastasis which will lead to intense pain, so far as to pathological fracture, which has tremendous reduction in the quality of life.^[2]^ The secretion of prostaglandin could accelerate the bone resorption around the tumor. Due to the intense sensibility of the nerve endings, sharp pain hence generates.^[3]^ According to vast research, it has proved that acupuncture could be efficient in analgesic therapy.^[4,5]^ Recent years have witnessed great promotion of the living standard, which also accompanied with the increasing morbidity of prostate cancer, and this has caused great impact on people's life and quality of life. Meanwhile, with the increasing progress of modern medicine, we could focus on not only prolonging lifespan of the cancerous person but also the impact to the quality of life.^[6,7]^ Enormous clinical literatures have indicated that acupuncture has positive effect on ameliorating pain and improve the quality of life in patients who suffer from advanced prostate cancer.^[8,9]^

Prostate cancer is the most common malignant tumor of the male genitourinary system, accounting for the fifth place in the global cancer rate. According to the WHO Global Cancer Epidemiology Statistics (GLOBOCAN 2008), the global morbidity of prostate cancer ranks second in males who suffer from malignancy in 2008 (ranking only second to lung cancer), accounting for 14%.^[10]^ Meanwhile, the morbidity also has significant difference across the world, which we found that the highest incidence, comparing with the lowest incidence, is about 25 times. In the United States, the morbidity of the disease ranks first in all male malignancies, accounting for about 29%. During 2004 to 2008, the morbidity of this cancer had been turned to 152.9 per 100,000, which was counted after the rise of age.^[11,12]^

Cancer pain is one of the most important symptoms in patients who suffer from advanced cancer; it greatly deteriorates the physical and psychological health and thus the quality of life of patients.^[13,14]^ The morbidity of the symptom is about 70% to 90%. According to the statistics that was announced by WHO in 2003, there are about 10 million new patients with cancer in the world every year, and 300 to 1000 million patients with cancer fail to receive timely and effective treatment.

At present, aiming at the symptom, the medical community mainly adopts the “three-step” analgesic drug therapy recommended by WHO, which means that the patients need long-term high-dose anesthetics to release the pain.^[15]^ Meanwhile, studies in the United States have found that using large doses of anesthetics for a long period of time is vulnerable to promoting tumor angiogenesis, accelerating tumor growth, and increasing the spread of cancer cells.^[16]^ Besides, patients who accept the treatment are always accompanied with constipation, nausea and vomiting, dizziness, drowsiness, rash, high blood pressure, coma, and other adverse reactions, so far, as to further aggravating the pain.^[17,18]^

As one part of traditional Chinese medicine (TCM), acupuncture therapy has been widely used in clinical trials of pain caused by prostate cancer in recent years.^[8]^ It can relieve pain caused by prostate cancer to some extent.^[9]^ On the basis of TCM theory, acupuncture can regulate the balance of qi and blood by stimulating acupuncture points, which aims at improving physiological function. Meanwhile, vast studies have shown that acupuncture tianshu (ST25), zusanli (ST36), and taichong (LR3) can adjust the production of neurotransmitter, mainly 5-hydroxytryptamine, and reduce nerve sensitivity.^[19]^ By preliminary database searching, we found that the randomized controlled trials about acupuncture for curing pain caused by prostate cancer have been increasing. However, most clinical trials confront with the inferior quality of the studies with small sample size and the insufficiency of evidence-based exploration because of the limitation of the size and number of clinical centers. Therefore, we expect to adopt meta-analysis to evaluate the efficacy and safety of acupuncture in the treatment of prostate cancer, to provide evidence for its clinical application.

Acupuncture has its unique advantages in the treatment of cancer pain. The operation is convenient; it could dredge the meridians and regulate the balance of qi and blood, which achieve the goal of relieving pain caused by qi stagnation and blood stasis and meridians impassability.^[20]^ The process of analgesia by acupuncture is complicated. Vast studies have proved that acupoint stimulation can accelerate the secretion of various mediators and opioid peptides from peripheral nerve to central nervous system, such as spinal cord, low brainstem, diencephalon, limbic system, and cerebral cortex, which together form the “anti-pain system” of the human body and produce acupuncture analgesic effects.^[21]^ With the deepening of the research and animal experiment on the mechanism of acupuncture and analgesia in recent years, there have been increasing reports on the application of acupuncture to various acute and chronic pain treatments.

## 2 Methods

This systematic review protocol has been registered on PROSPERO as CRD42018111550 (http://www.crd.york.ac.uk/PROSPERO/display_record.php?ID=CRD42018111550) The protocol follows the Cochrane Handbook for Systematic Reviews of Interventions and the Preferred Reporting Items for Systematic Reviews and Meta-Analysis Protocol (PRISMA-P) statement guidelines. We will describe the changes in our full review, if needed.

### 2.1 Inclusion criteria for study selection

#### 2.1.1 Types of studies

The selected literature will include the acupuncture-related randomized controlled trials that aim at treating pain caused by prostate cancer. The language is limited to Chinese and English. Nonrandomized controlled trials, quasi-randomized controlled trials, case series, case reports, and crossover studies will be excluded.

#### 2.1.2 Types of participants

Male patients who were diagnosed with prostate cancer will be included; the type of the disease includes adenocarcinoma (adenocarcinoma), ductal adenocarcinoma, urothelial carcinoma, squamous cell carcinoma, and adenosquamous carcinoma. Meanwhile, the patients who were recruited should have the symptom of pain caused by prostate cancer, including bone pain caused by bone metastasis. However, the patients who suffer from pain caused by other factors would be excluded. Patients who suffer from cognition impairment and other severe mental illness will also be excluded. In addition, the inclusion criteria will not be restricted by region, country, nation, and origin.

#### 2.1.3 Types of interventions

##### 2.1.3.1 Experimental interventions

The patients who were included by experimental group should accept the conventional treatment and acupuncture therapy that includes conventional acupuncture, electropuncture, ignipuncture, plum-blossom needle, and massaging acupoints. Pharmacoacupuncture and acupoint injection will be rejected, as their methods and theories are different from TCM. The duration and frequency of therapy are not limited.

##### 2.1.3.2 Control interventions

The patients who belong to control group should be treated with conventional therapy or combined with simple analgesics. However, once they had accepted acupuncture combined with medication or other therapy of TCM, the trials will be rejected.

The following treatment comparisons will be investigated:

1. Acupuncture versus no treatment
2. Acupuncture versus placebo/sham acupuncture
3. Acupuncture versus drug therapy
4. Acupuncture versus other active therapies
5. Acupuncture with another active therapy versus the same therapy alone

#### 2.1.4 Types of outcome measures

##### 2.1.4.1 Primary outcomes

Adopting numerical rating scale (NRS) of pain criteria as the main evaluation, the specific division are as follows: painless 0 points; mild pain 1 to 3 points; moderate pain 4 to 6 points; severe pain 7 to 9 points; and severe pain 10 points, 0 to 10 points representing the patient's pain level. “Significantly effective” denotes that the patient's pain level is reduced by at least two or three levels, or the patient is painless; “Effective” denotes that the patient's pain level is reduced by one grade or the patient presents moderate and mild pain; and “Invalid” denotes that the patient's pain does not have any relief, and there is even a tendency to aggravate.

##### 2.1.4.2 Secondary outcomes

Secondary outcomes are as follows:

1. International prostate symptom score, the patient self-evaluation form;
2. Quality-of-life scores for patients with prostate cancer before and after treatment: A scale, designed by the University of California, USA, to evaluate the condition and living quality of patients who suffer from advanced, hormone-insensitive prostate cancer. It specifically includes nine aspects: physical strength, pain, fatigue, appetite, family/marriage, constipation, mood, defecation, and overall feelings. Each item is calculated with 100 points, and the lower the score, the worse the situation.

### 2.2 Search methods for the identification of studies

#### 2.2.1 Electronic searches

Database search: Searching PubMed, Cochrane Library, AMED, EMbase, WorldSciNet, Nature, Science online and China National Knowledge Infrastructure (CNKI), China Biology Medicine disc (CBM), and China Resources Database. The temporal interval is limited from the time that the databases created to November 2018, and the combination of keyword and free word retrieval is adopted. The search terms include “acupuncture,” “acupuncture,’, “electro-acupuncture,” “fire needle,” “plum blossom needle,” “skin needle,” “prostate cancer,” “prostate cancer,” “pain,” “Random Control.”

#### 2.2.2 Searching other resources

We will search ongoing experimental studies related to the disease through the WHO International Clinical Trial Registration Platform, ClinicalTrials.gov, and Chinese clinical trial studies. As for these ongoing experiments, we will try to contact the trial author to help provide the most up-to-date clinical data. Besides, we also tempt to adopt the manual searching which mainly aims at relevant literatures, earlier than the database mentioned above, such as “China Rehabilitation Medicine Journal,” “Chinese Acupuncture,” “Chinese Journal of Physical Medicine and Rehabilitation,” “Acupuncture Clinical Journal,” and “Chinese Journal of Urology.” Search strategy for Medline is shown in Table 1.

**Table 1:**
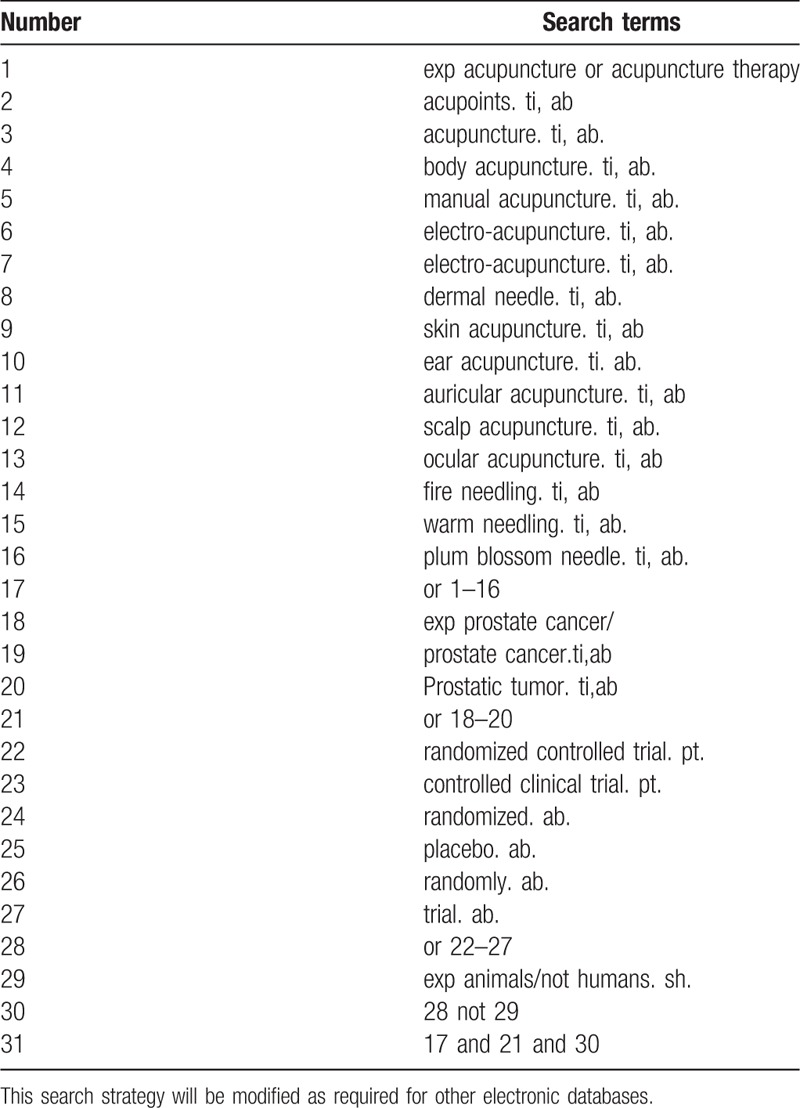
Search strategy used in PubMed database.

### 2.3 Data collection and analysis

#### 2.3.1 Selection of studies

**Figure 1:**
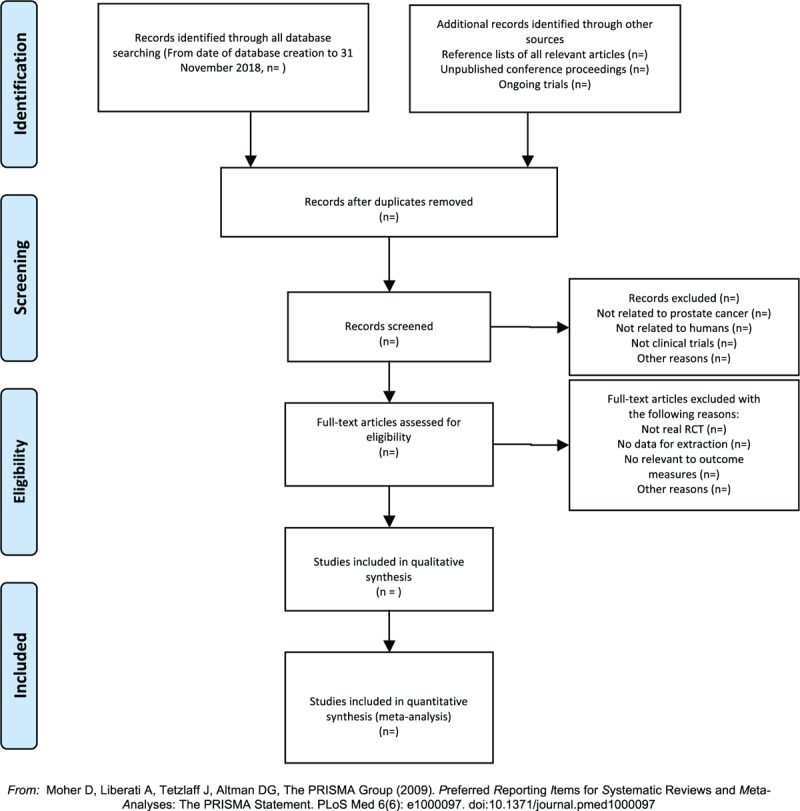
The PRISMA flow chart.

Initial evaluations were independently performed by two investigators through filtrating the titles and abstracts of each documents in the Endnote database, eliminating duplicates and documents that were clearly inconsistent with the study. After the preliminary assessment, to screen out eligible trials, the full text of the selected literature would be evaluated, which mainly aims at whether there were problems just like uncontrolled studies, no randomization, inconsistent assessment criteria, and similar data. When the two researchers could not reach a consensus, the third judge would make the final judgment. The primary selection process is shown in a PRISMA flow chart (Fig. 1).

#### 2.3.2 Data extraction and management

There will be two investigators independently extracting information from the included literature, mainly contents that are author (year), sample size, disease stage, patient age, intervention factors, control factors, intervention time, observation index, NRS score, symptoms ratings, quality-of-life scores, etc. The extracted literature data is filled in a unified data statistics table.

#### 2.3.3 Assessment of risk of bias in included studies

Two investigators independently assess the quality of the included literature by using the Cochrane Collaboration's bias risk assessment tool. The assessment includes random sequence generation, allocation concealment, blinding, incomplete outcome data, selective outcome reporting, and other possible biases. According to the relevant standards in the Cochrane Intervention System Evaluation Manual, it is divided into low risk, high risk, and unclear.

#### 2.3.4 Measures of treatment effect

For continuous variable outcome, mean difference (MD) or standardized mean difference (SMD) and 95% confidence interval (95% CI) will be recorded. For dichotomous outcomes, we will use the relative risk (RR) and 95% CI records.

#### 2.3.5 Unit of analysis issues

We will only extract the first experimental period data of crossover trials to avoid carryover effects. With multiple intervention groups, we will combine all similar experimental groups and control groups into 1 group to prevent a unit of analysis issue.

#### 2.3.6 Dealing with missing data

In the event of data loss during the screening and extraction of literature data, primarily, we would actively look for the cause of the loss, and then we would contact the experimental research author by telephone, mail, etc., to retrieve the lost data. If the loss could not be retrieved, we will only extract and analyze the useful data, and the situation would be indicated.

#### 2.3.7 Data synthesis and analysis

The analysis of the data will adopt RevMan 5.3 software. As for the two categorical variables, we select RR or odds ratio (OR) and 95% CI. As for the continuous variables, we select weighted mean difference (WMD) or standard mean difference (SMD) and 95% CI, the difference would be statistically significant when *P* < 0.05. Heterogeneity test would be analyzed by using chi-square test. When *P* ≥ 0.1, the difference was considered to be not statistically significant. When *P* < 0.1, *I*^2^ > 50%, the random-effect model would be used, as for the other situation, the fixed-effect model would be adopted.

#### 2.3.8 Assessment of heterogeneity

If there is significant heterogeneity between a group of studies, we will explore the reasons for the existence of heterogeneity from various aspects, such as the characteristics of the patients and the degree of variation of the interventions. Necessarily, sensitivity analysis or subgroup analysis would be adopted to explain the heterogeneity.

#### 2.3.9 Assessment of publication bias

The forest map and funnel plot would be drawn and analyzed by RevMan 5.3 software, and the funnel plot would be used to analyze the potential publication bias.

#### 2.3.10 Sensitivity analysis

We will perform sensitivity analysis for primary outcomes to test the robustness of the review conclusions, and we will still evaluate the impact of methodological quality, sample size, and missing data.

#### 2.3.11 Grading the quality of evidence

The quality of evidence for the main outcomes will also be assessed with the Grading of Recommendations Assessment approach. The evaluation included bias risk, heterogeneity, indirectness, imprecision, and publication bias. And each level of evidence will be made “very low,” “low,” “erate,” or “high” judgment.

## 3 Discussion

At present, pain has been the fifth vital sign, together with respiration, blood pressure, pulse, and body temperature. In 1996, the American Pain Association first proposed the concept of pain and its control, which indicated that an effective pain relief program for patients is a basic requirement for clinical medical work. It has been a difficulty problem to the treatment of pain caused by prostate cancer and its bone metastasis in the middle and advantaged stage, which has belonged to refractory cancer pain.^[22]^ Recent years have witnessed an increase in the study of treating cancer pain; vast researches have indicated that analgesics have great efficiency in ameliorating pain, but the pesticide effects are short, and there are kinds of adverse reaction, such as kidney damage, liver damage, cardiovascular and cerebrovascular damage, and digestive disease.^[23,24]^ Generally speaking, numerous patients who suffer from cancer could not gain satisfied control effect. Moreover, these patients need long-term use of analgesic drugs to ameliorate pain, which also directly increase the cost of therapy for patients.

Acupuncture, possessing thousand years of history in China, has proved that it is a safe, feasible, and effective treatment. The clinical operation and theory of acupuncture have been inherited and carried forward by the majority of clinicians, especially in the treatment of pain. This therapy could ameliorate vast kinds of pain efficiently, and the selection of acupoint is convenient, besides, there are not adverse reaction which could insure the security.^[25]^ In recent years, acupuncture therapy has been widely used in clinical trials of pain caused by prostate cancer, and the recent studies have shown that the therapy could ameliorate the pain caused by prostate cancer to some extent and improve the quality of the life.^[26]^

As far as we know, there has no comparison made of the effectiveness of acupuncture in the treatment of pain caused by prostate cancer. Therefore, we will use systematic review and meta-analysis to evaluate the efficacy and safety of acupuncture for the treatment of pain caused by prostate cancer. We expect that the review could provide a basis for acupuncture treatment of pain caused by prostate cancer and offer more and better options for treatment to patients. In addition, the literature on acupuncture therapy on pain caused by prostate cancer is relatively inadequate and the overall quality is a little low, which may affect the authenticity of this study.

## Author contributions

**Data curation:** Jisheng Wang, Yi Lei, Fei Chen.

**Formal analysis:** Jisheng Wang, Binghao Bao.

**Funding acquisition:** Haisong Li.

**Project administration:** Bin Wang.

**Supervision:** Haisong Li, Bin Wang.

**Validation:** Haisong Li.

**Writing – original draft:** Jisheng Wang, Yi Lei.

**Writing – review & editing:** Xudong Yu, Hengheng Dai.
